# Roles of Klf5 Acetylation in the Self-Renewal and the Differentiation of Mouse Embryonic Stem Cells

**DOI:** 10.1371/journal.pone.0138168

**Published:** 2015-09-15

**Authors:** Tong Zhao, Chang Liu, Lingyi Chen

**Affiliations:** 1 State Key Laboratory of Medicinal Chemical Biology, Collaborative Innovation Center for Biotherapy, 2011 Collaborative Innovation Center of Tianjin for Medical Epigenetics and College of Life Sciences, Nankai University, Tianjin, China; 2 State Key Laboratory of Molecular Oncology, Cancer Institute/Hospital, CAMS, Beijing, China; National University of Singapore, SINGAPORE

## Abstract

Transcription factor Krüppel-like factor 5 (Klf5) plays important roles in the formation of the inner cell mass (ICM) and the trophectoderm during embryogenesis, as well as the self-renewal and the differentiation of mouse embryonic stem cells (ESCs). Acetylation of KLF5 has been shown to reverse the transcriptional activity of KLF5 in human epidermal cells and prostate cancer cells. Whether Klf5 acetylation contributes to the lineage specification in the blastocyst and pluripotency maintenance in ESCs remains unexplored. Here, we showed the ubiquitous expression of acetylated Klf5 in the ICM and the trophectoderm, ruling out the possibility that differential acetylation status of Klf5 leads to the lineage specification in the blastocyst. We found that K358Q mutation, mimicking acetylation, enhances the transcriptional activity of Klf5 for pluripotency genes in ESCs, and that K358Q Klf5 is more potent in pluripotency maintenance and in somatic cell reprogramming, compared to K358R Klf5. In ESCs, Klf5 acetylation, stimulated by TGF-β signaling, is involved in enhancing Sox2 expression. Moreover, upon ESC differentiation, acetylation of Klf5 facilitates the suppression of many differentiation genes, except for that K358Q Klf5 activates *Cdx2*, promoting trophectodermal differentiation. In summary, our results revealed the regulatory functions of Klf5 acetylation in ESC self-renewal and differentiation.

## Introduction

During mouse preimplantation development, two distinct cell lineages, the inner cell mass (ICM) and the trophectoderm (TE), are first established in the blastocyst. The pluripotent ICM cells mainly contribute to the development of the fetus, while the TE develops into the placenta [1, 2]. In addition, under proper *in vitro* culture conditions, the ICM gives rise to embryonic stem cells (ESCs). ESCs self-renew indefinitely and maintain the potential to differentiate to all cell types in the body [3, 4]. Thus, ESCs have great application potential in regenerative medicine. Understanding the molecular mechanisms underlying ESC self-renewal and pluripotency is critical to the field of regenerative medicine.

It has been revealed that a unique transcriptional regulation network is essential for pluripotency maintenance of ESCs [5, 6]. Oct4, Sox2 and Nanog, forming a feed-forward self-regulating circuitry, are the core components of the transcriptional regulation network for pluripotency [7–10]. Krüppel like factors, Klf2, Klf4 and Klf5, are also key players in the transcriptional regulation network of pluripotency [11–16]. They are highly expressed in mouse ESCs, and down-regulated upon differentiation. These three Krüppel-like factors share redundant functions in pluripotency maintenance and establishment. ESC colony morphology and alkaline phosphatase (AP) positivity are lost upon simultaneous knockdown of all three Klf factors, while knockdown of individual Klf factor does not affect ESC colony morphology or AP positivity [11]. Both Klf2 and Klf5 can substitute for Klf4, together with Oct4, Sox2 and c-Myc, to reprogram somatic cells [17]. Moreover, the genomic binding profiles of these three Klf factors are highly overlapped [11].

However, other evidences suggest that Klf5 has unique roles in pluripotency regulation. In contrast to Jiang’s work mentioned above [11], others have demonstrated that knockdown or knockout of Klf5 alone leads to down-regulation of pluripotency gene expression, as well as spontaneous ESC differentiation [13, 15]. The conflicting results might be explained by the differences in knockdown efficiency and experimental settings. During ESC differentiation, Klf5 suppresses mesodermal differentiation, while Klf4 represses the expression of endodermal genes [18]. All these data suggest that Klf5 is essential for the maintenance of pluripotency in ESCs, and its function cannot be fully compensated by Klf2 and Klf4. In addition to its role in pluripotency maintenance, Klf5 is also involved in TE development. Klf5 is expressed in the ICM and the TE, as well as their *in vitro* counterparts, ESCs and trophectoderm stem cells (TSCs). In contrast, Klf2 and Klf4 are only expressed in ESCs, but not in TSCs. Consistently, *Klf5* knockout embryos die before implantation and have defects in both the ICM and the TE. No ESC lines can be derived from *Klf5* knockout ICM cells. Knockout of *Klf5* also reduces the expression of *Cdx2*, a key transcription factor for TE development, in TE cells, resulting in implantation failure [14, 19]. Klf5 is required for the expression of *Cdx2* in TE cells, while it activates pluripotency gene expression in ESCs. How the transcriptional activity and downstream targets of Klf5 is regulated in these two distinct cell types, remains elusive.

It has been reported that the transcriptional activity of Klf5 is regulated by various post-transcriptional modifications, such as phosphorylation, acetylation, ubiquitination and sumoylation [20–27]. The expression level of KLF5 is tightly regulated by the ubiquitin-proteasome system. And WWP1 has been identified as an E3 ubiquitin ligase for KLF5 [24]. Phosphorylation of KLF5 enhances its interaction with CREB binding protein (CBP), and hence increases its transcriptional activity [21]. Sumoylation may regulate the cytoplasmic and nuclear localization of KLF5 [26]. In addition, both sumoylation and acetylation of KLF5 affects the transcriptional complexes that KLF5 associates with, switching transcriptional activity of KLF5 between activator and repressor [20, 27, 28]. Most strikingly, in the HaCaT epidermal cell line, transforming growth factor-β (TGF-β) mediated acetylation of human KLF5 at lysine 369 reverses the transcriptional activity of KLF5, from a transcriptional repressor to a transcriptional activator for the *p15* (*CDKN2B*) gene. Consequently, the pro-proliferative factor KLF5 becomes anti-proliferative upon activating TGF-β signaling [20, 28]. The opposing function of KLF5 regulated by K369 acetylation has also been demonstrated in prostate tumorigenesis [29].

Given the critical regulatory function of KLF5 acetylation, we investigated the role of Klf5 acetylation in the differentiation of the ICM and the TE, as well as in maintaining the pluripotency of mouse ESCs. We first demonstrated that Klf5, unacetylated (unAc-) and acetylated (Ac-) Klf5 are expressed in both ICM and TE cells, at similar levels. It implies that the segregation of the ICM and the TE is not caused by differential level of Ac-Klf5. Next, we showed that the K358Q (KQ) Klf5 mutant, mimicking Ac-Klf5, promotes ESC self-renewal more efficiently than the K358R Klf5 mutant (KR, mimicking unAc-Klf5). Acetylation of Klf5, regulated by TGF-β signaling, increases Sox2 protein expression. In addition, during ESC differentiation, KQ Klf5, but not KR Klf5, is able to suppress the expression of differentiation genes, except for *Cdx2*. KQ Klf5 facilitates the activation of *Cdx2* in embryoid bodies (EBs), implying that Klf5 acetylation might be required for the TE development *in vivo*. All these data suggest a regulatory role of Klf5 acetylation in the self-renewal and the differentiation of mouse ESCs.

## Materials and Methods

### Ethics statement

This study was carried out in strict accordance with the recommendations in the Guide for the Care and Use of Laboratory Animals of the National Institutes of Health. The protocols and the use of mice for this research were approved by Nankai Animal Care and Use Committee.

### Cell culture

ESCs and TSCs were cultured as described previously [30]. In brief, V6.5 mouse ESCs were cultured in growth medium consisting of 85% DMEM (Invitrogen), 15% fetal bovine serum (Hyclone), 2 mM L-glutamine, 5000 units/ml penicillin and streptomycin, 0.1 mM non-essential amino acids (Invitrogen), 0.1 mM 2-mercaptoethanol (Sigma), and 1000 units/ml LIF (Chemicon). TSCs were cultured in TSC culturing medium containing 80% RPMI 1640, 20% fetal bovine serum (Hyclone), 25 ng/ml Fgf4 (Sigma), 1 ng/ml heparin (Sigma), 0.1 mM 2-mercaptoethanol (Sigma), 1 mM sodium pyruvate (Invitrogen), 2 mM L-glutamine (Invitrogen), 5000 units/ml penicillin and streptomycin (Invitrogen).

### Embryo culture

Female ICR mice (4–6 weeks) were induced to superovulate by intraperitoneal injections of 5 IU of pregnant mare serum gonadotropin (PMSG, Calbiochem) and 48 h later 5 IU human chorionic gonadotropin (hCG, Sigma). Then females were paired with ICR males overnight and checked for vaginal plugs the following morning. Two-cell embryos were flushed from oviducts at 42–48 h post-hCG and cultured in groups of 20–30 in a 50 μL drop of potassium simplex optimization medium (KSOM) with amino acids (Millipore) covered by mineral oil (Sigma, for embryo culture) in a 37°C incubator with 6.5% CO_2_. All experiments were performed with groups of more than 10 embryos and repeated three times.

### Immunofluorescence

E3.5 embryos were fixed in 4% paraformaldehyde for 20 min, and then permeabilized with 0.2% Triton X-100 for 30 min. After being blocked with 5% goat serum for 2 h, embryos were incubated with primary antibodies for 4–6 h at room temperature or overnight at 4°C. Then embryos were washed and incubated with secondary antibodies. We used the following primary antibodies: Cdx2 antibody (Biogenex), total Klf5, Ac-Klf5, and unAc-Klf5 antibodies (gifts from Dr. Jin-Tang Dong)[20]. We used Alexa Fluor 488 anti-rabbit and Alexa Fluor 594 anti-mouse as secondary antibodies (Molecular Probe), and Hoechst 33342 (Sigma) for nuclei staining. Confocal images were captured using Leica TCS SP5 confocal microscope.

### Generation of stable Klf5 knockdown or overexpression ESC Lines

Gene knockdown was performed with the pSuper-puro system (Oligoengine) following the manufacturer’s instruction. The 19-nt targeting sequences for Klf5 was GACCATGCGTAACACAGAT. WT, KR and KQ Klf5 were cloned into the pCAGIPuro expression plasmid (a gift from Dr. Hitoshi Niwa). Empty pCAGIPuro, WT and mutant Klf5 overexpression, shGFP, and shKlf5 plasmids were transfected into V6.5 ESCs with Lipofectamine 3000. After 7- to 10-day puromycin or hygromycin selection, single colonies were picked, and knockdown or overexpression efficiencies were determined by quantitative RT-PCR.

### Colony forming assay

Mouse ESCs (500 cells per well of a 6-well plate) were cultured under differentiation condition (without feeders and LIF) for four days. Cells were washed once with PBS solution and incubated with alkaline phosphatase substrate kit III (Vector) for 20 min at room temperature. AP-positive colony number was counted under the microscope.

### Embryoid body differentiation

To form EBs, ESCs were cultured in 30 μL hanging drops (1000 cells/drop, ESC medium without LIF), and EBs were collected on day 4.

### Quantitative RT-PCR

Total RNA was extracted from cells using the RNeasy mini kit (Qiagen). cDNA synthesis was performed using the Tanscriptor First Strand cDNA Synthesis Kit (Roche) with random primers according to the manufacturer’s instruction. PCR reactions were performed with FastStart Universal SYBR Green Master (Roche) in a Bio-Rad iQ5 system. PCR cycling conditions were 95°C for 2 min, 40 cycles of 95°C for 15 s, 58°C for 15 s, and 72°C for 30s, and then a melting curve of the amplified DNA was acquired. Quantification of target genes was normalized to β-Actin. Primer information was listed in S1 Table.

### Western blot

Cells were lysed in lysis buffer (Beyotime), and protein concentration was measured using a BCA protein assay kit (Beyotime) to ensure equal loading. The samples were resolved by SDS-PAGE, followed by transferring onto a PVDF membrane (Millipore). Membranes were probed with anti-Sox2, anti-Klf5 (GeneTex), anti-β-Tubulin (Huada), anti-Oct3/4 (Santa Cruz Biotechnology), anti-Nanog (Bethyl Laboratories), anti-Flag (sigma), and anti-Klf5 antibodies from Jin-Tang Dong’ lab. Bound primary antibodies were recognized by HRP-linked secondary antibodies (GE Healthcare). Immunoreactivity was detected by ECL Plus (Beyotime). Digital images were taken with by the automatic chemiluminescence imaging analysis system (Tanon). The intensity of bands was quantified with the Quantity One analysis software (Bio-Rad).

### Reprogramming assay

To pack retroviruses, pMXs-based retroviral vectors (pMXs-Sox2, Oct4, c-Myc, as well as WT and mutant Klf5) were introduced into Plat-E cells using Lipofiter transfection reagent (Hanheng) according to the manufacturer's protocol. MEFs were infected twice with different combination of viruses on day 0 and 2. After the second viral infection, the cells were cultured in ESC medium, and medium was changed every day. On day 14, the number of iPS colony was counted under microscope.

### Statistical analysis

Data were analyzed by two-tailed Student's t test unless specified in figure legends. When reusable two-factor analysis of variance was performed, it was indicated in figure legends. Statistically significant p values were indicated in figures as follows: **, p < 0.01; *, p < 0.05. Averages and standard deviations of at least three independent experiments were shown in figures when it is applicable.

## Results

### The expression levels and acetylation levels of Klf5 are similar in the ICM and the TE

**Fig 1:**
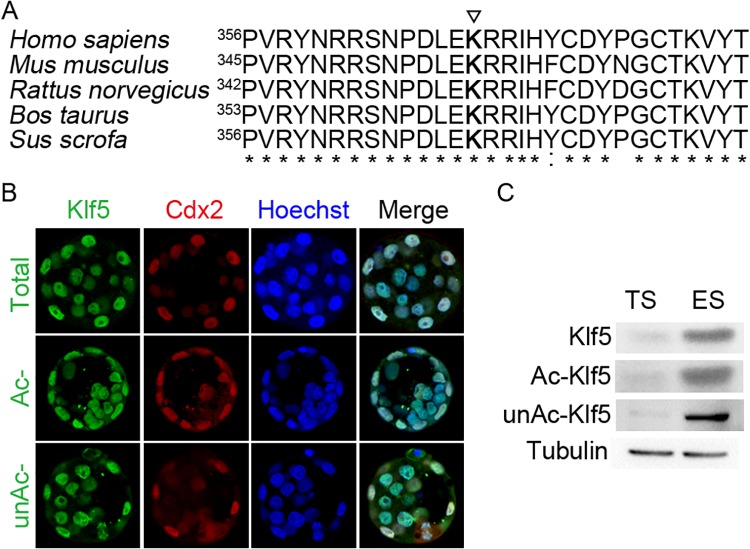
Klf5 expression in the blastocyst, ESCs and TSCs. (A) Comparison of different species Klf5 protein sequences around the human KLF5 acetylation site. The numbers mark the coordination of the first shown residues “P” in Klf5 proteins of different species. The triangle highlights the conserved lysine residue 369 that is acetylated in human KLF5. (B) Immunofluorescence staining of total, acetylated (Ac-), and unacetylated (unAc-) Klf5 in the blastocyst. Cdx2 expression was shown to mark TE cells, and nuclei were stained by Hoechst. (C) Western blot of total, Ac-, and unAc-Klf5 in mouse ESCs and TSCs. Tubulin was used as a loading control.

Acetylation of human KLF5 at lysine 369 switches its transcriptional activity from a repressor to an activator for the *p15* gene in the HaCaT epidermal epithelial cell line and prostate cancer cells [20, 28, 29]. Whether this acetylation site is conserved in other species, and plays critical regulatory function in other biological processes, such as the segregation of the ICM and the TE, remains to be explored. We first analyzed the homology of the Klf5 proteins across different species. Indeed, Klf5 is highly conserved among human, chimpanzee, Rhesus monkey, dog, cow, mouse, rat, chicken, zebrafish, and frog. For example, human and mouse Klf5 share 87% identity. Moreover, peptide sequences around the known acetylation site of human KLF5 (K369) have even higher homology across species, almost identical for human, mouse, rat, cow, and pig Klf5 (Fig 1A). The highly conserved sequence at the acetylation site implies that the regulatory function of Klf5 acetylation is likely conserved in these species. In addition, through the sequence alignment, the lysine residue 358 of mouse Klf5 protein is identified as a potential acetylation site.

We then tested whether Klf5 has differential acetylation status in ICM and TE cells, leading to the segregation of these two types of cells? We examined the expression of Klf5, Ac- and unAc-Klf5) in the blastocyst by immunofluorescence. Consistent with previous reports [14, 19], Klf5 is ubiquitously expressed in the blastocyst. In addition, no biased expression of Ac-Klf5 and unAc-Klf5 in ICM and TE cells was observed (Fig 1B). We further examined the expression of Klf5, Ac- and unAc-Klf5 in ESCs and TSCs, the *in vitro* counterparts of the ICM and the TE. Again, all three forms are present in ESCs and TSCs. Even though ESCs express more Klf5 than TSCs, the ratios of Ac-Klf5 to total Klf5 in ESCs and in TSCs are about the same (Fig 1C). The similar acetylation levels of Klf5 in the ICM and the TE, as well as their *in vitro* counterparts, rule out the possibility that differential acetylation status of Klf5 facilitates the differentiation of the ICM and the TE.

### Klf5 acetylation promotes the self-renewal of mouse ESCs

**Fig 2:**
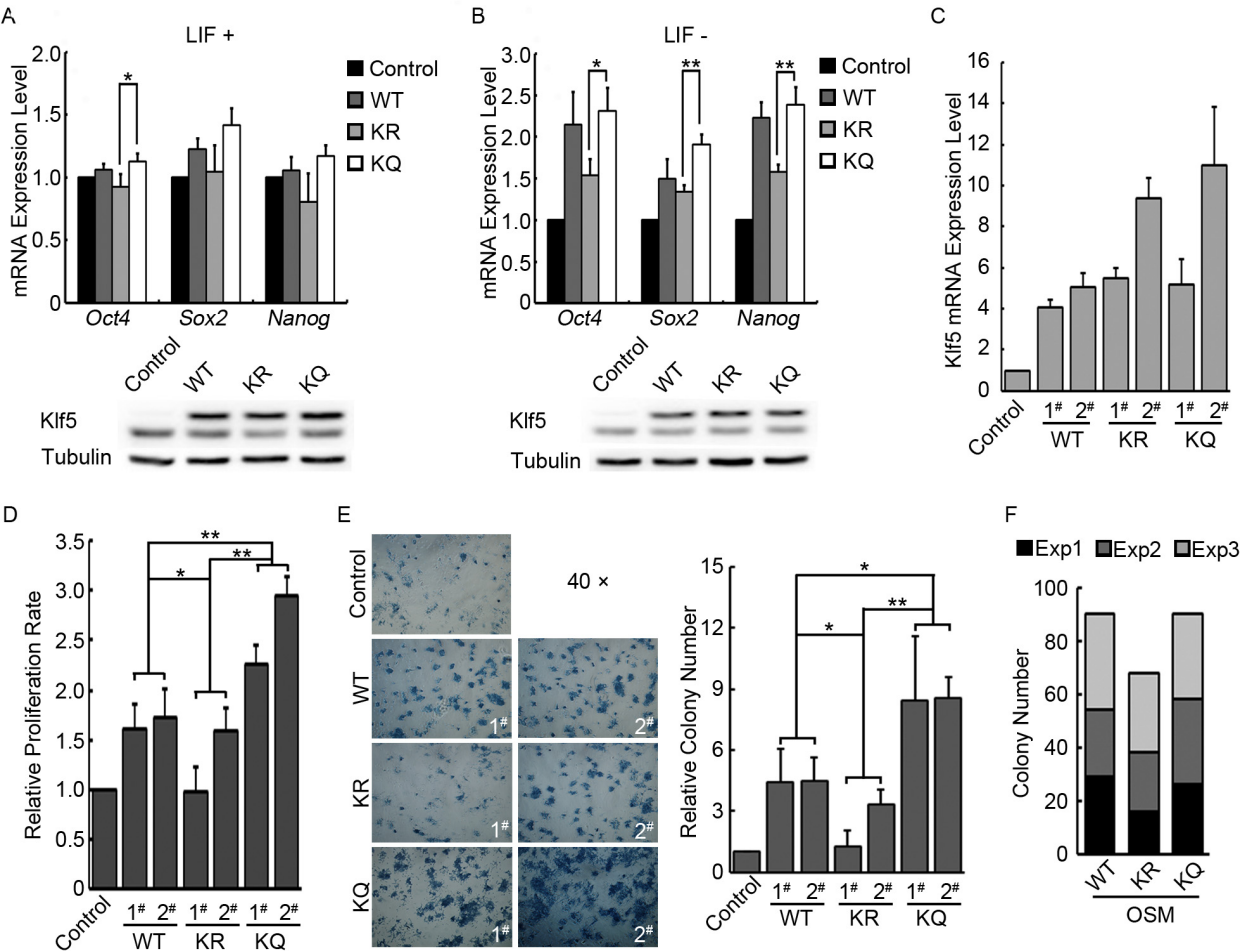
Functions of Ac-Klf5 in self-renewal and pluripotency regulation. (A) The effect of WT, KR, and KQ Klf5 overexpression on the expression of pluripotency genes in mouse ESCs. Empty pCAGIPuro, Flag-tagged WT, KR and KQ Klf5 overexpression plasmids were transfected into V6.5 ESCs with Lipofectamine 3000. Two days after transfection, cells were harvested and subjected to RNA purification or Western blot. The top panel shows RNA quantification results (averages and standard deviations from three independent experiments were plotted), and the bottom panel shows Western blots to demonstrate the similar expression levels of Flag-tagged WT, KR and KQ Klf5. (B) The effect of WT, KR, and KQ Klf5 overexpression on the expression of pluripotency genes during mouse ESC differentiation. Experiments were carried out as described in (A), except for that LIF was withdrawn from the medium upon transfection. Averages and standard deviations from three independent experiments were plotted. (C) Relative expression level of total Klf5 mRNA in control, WT, KR, and KQ Klf5 overexpression ESC lines. Averages and standard deviations from three independent experiments were plotted. (D) Proliferation rates of control, WT, KQ, and KR Klf5 overexpression ESCs. 1.5×10^5^ Cells were seeded in one well of a 6-well plate, and 48 hours later, total cell number was counted to measure the proliferation rate. Averages and standard deviations from three independent experiments were plotted. P values were calculated by reusable two-factor analysis of variance. (E) Colony forming assays for control, WT, KR, and KQ Klf5 overexpression ESCs growing without LIF and feeder cells. The left panel shows the image of AP stained colonies of each ESC line, and the quantification result of colony number is plotted in the right panel. Averages and standard deviations from three independent experiments were plotted. P values were calculated by reusable two-factor analysis of variance. (F) Reprogramming ability of WT, KR, and KQ Klf5 in combination with Oct4, Sox2 and c-Myc. MEFs were reprogrammed by WT, KQ, or KR Klf5, together with Oct4, Sox2, and c-Myc. Accumulated iPS colony numbers of three independent experiments (labeled with three different colors) are shown in the plot.

We have detected acetylated Klf5 in mouse ESCs. Given the important role of Klf5 in pluripotency regulation, we then asked how acetylation of Klf5 affects its transcriptional activity and its function in pluripotency maintenance. To this end, the lysine residue 358 of mouse Klf5 was replaced by an arginine residue (KR mutant) to mimic the unacetylated status, or by a glutamine residue (KQ mutant) to mimic the acetylated status. Plasmids expressing WT, KR and KQ Klf5 were transfected into ESCs, and the expression of key pluripotency genes, *Oct4*, *Sox2*, and *Nanog*, were measured by quantitative RT-PCR. However, we did not observe significant activation of these key pluripotency genes by any type of Klf5 in ESCs. Nevertheless, it seems that KQ Klf5 is more potent in activating the transcription of these key pluripotency genes, than KR Klf5 (Fig 2A). It is possible that endogenous level of Klf5, as well as other activators, activate the key pluripotency genes to a saturation level in undifferentiated ESCs, thus masking any difference among WT, KR, and KQ Klf5. To better detect the functional difference of WT and mutant Klf5, we repeated the overexpression experiment, except for that LIF was withdrawn from the ESC medium upon transfection to allow spontaneous ESC differentiation. Under this experimental condition, the activation effect of Klf5 on *Oct4*, *Sox2*, and *Nanog* became obvious. More importantly, WT and KQ Klf5 activate the key pluripotency genes more efficiently, compared to KR Klf5. And Western blot demonstrated the similar expression levels of WT, KR and KQ Klf5 (Fig 2B). These data suggested that acetylation of Klf5 enhances its transcriptional activity in ESCs.

We then asked whether acetylation of Klf5 regulates the proliferation and the self-renewal of ESCs. Stable ESC lines overexpressing WT, KQ, and KR Klf5 were first constructed, and the expression levels of *Klf5* in different cell lines were quantified (Fig 2C and S1 Fig). While culturing these cell lines, we noticed significant variation of proliferation rates. At a similar expression level of *Klf5* (WT 1# and 2#, KQ 1#, and KR 1#), KQ Klf5 ESCs proliferates faster than ESCs expressing WT and KR Klf5. Even when the variation of Klf5 expression level was taken into account, reusable two-factor analysis of variance revealed that the effects of WT, KR, and KQ Klf5 on ESC proliferation are significantly different. Moreover, the proliferation rate of ESCs is correlates with *Klf5* expression level. The clones expressing more *Klf5* (KQ 2# and KR 2#) grow faster than the clones expressing less *Klf5* (KQ 1# and KR 1#), respectively (Fig 2D). A similar result was also observed in colony forming assays. KQ Klf5 ESCs form more AP positive colonies than ESCs expressing WT and KR Klf5, while KR Klf5 ESCs give rise to the least AP positive colonies (Fig 2E).

Now we have demonstrated that acetylation of Klf5 facilitates the activation of key pluripotency genes, and promotes ESC proliferation and self-renewal. Does Ac-Klf5 outperform unAc-Klf5 in somatic cell reprogramming? To address this question, WT, KQ, or KR Klf5, together with Oct4, Sox2, and c-Myc, were used to reprogram mouse embryonic fibroblasts (MEFs). It appears that reprogramming with WT and KQ Klf5 yields more induced pluripotent stem (iPS) cell colonies, compared to reprogramming with KR Klf5, even though the difference is not statistically significant (Fig 2F).

### TGF-β signaling activates acetylation of Klf5 in mouse ESCs

**Fig 3:**
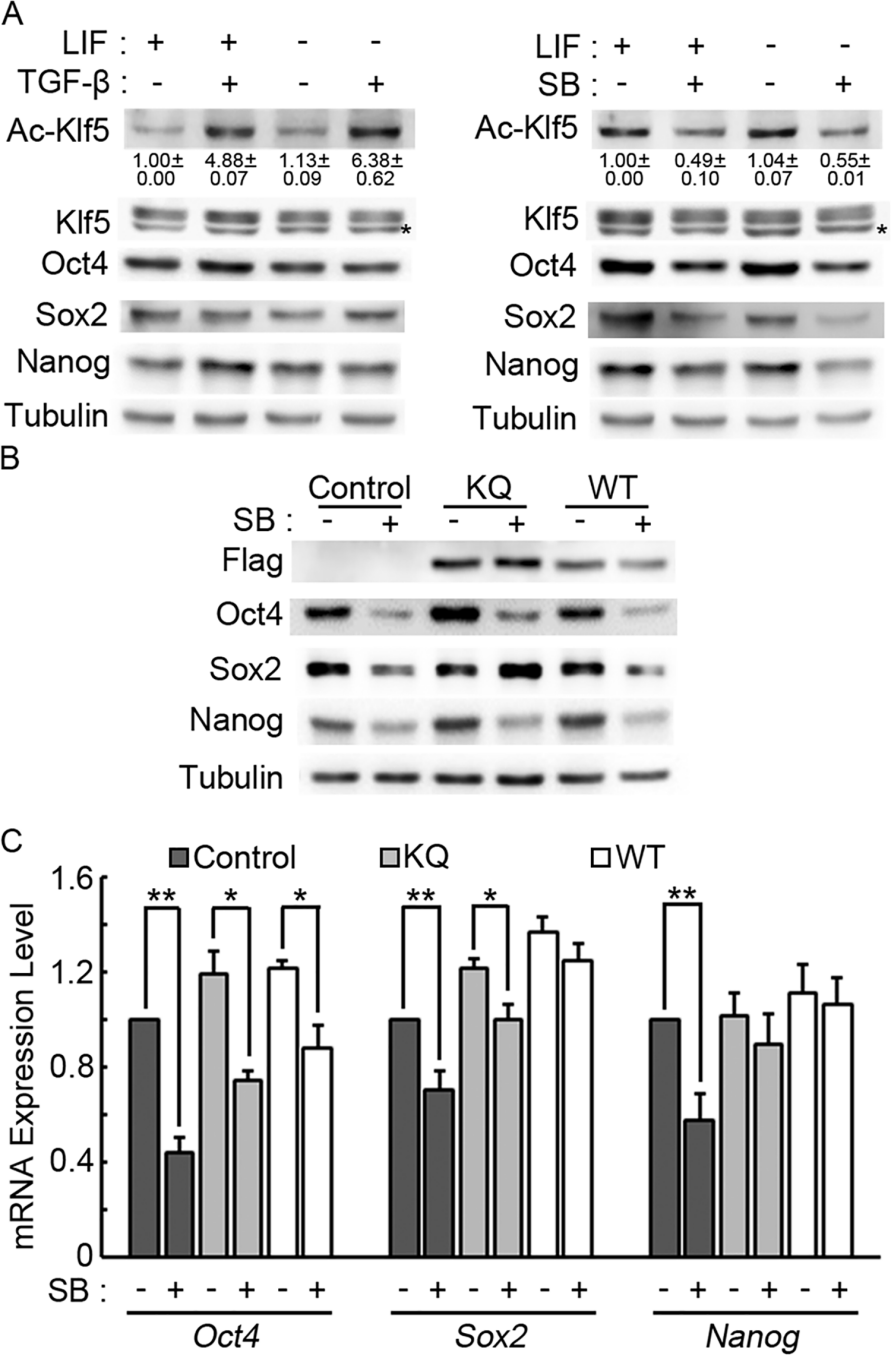
Klf5 acetylation, stimulated by TGF-β signaling, facilitates pluripotency maintenance in mouse ESCs. (A) TGF-β signaling promotes Klf5 acetylation. V6.5 ESCs were treated with 10 μg/ml TGF-β or 5 μM SB525334 (SB) for 48 hours, and then harvested for subsequent Western blot experiments. The nonspecific band in the anti-Klf5 blot is marked by an asterisk. The expression levels of Ac-Klf5 were quantified and normalized to Klf5. Averages and standard deviations of Ac-Klf5 expression from three experiments are shown. (B) Overexpression of KQ Klf5 mutant antagonizes the suppression effect of TGF-β signaling on Sox2 protein expression. Empty pCAGIPuro, Flag-tagged WT and KQ Klf5 overexpression plasmids were transfected into V6.5 ESCs. Cells were cultured in mouse ESC medium with or without 5 μM SB525334 for 2 days, and then harvested for subsequent Western blot experiments. The nonspecific band in the anti-Klf5 blot is marked by an asterisk. (C) The effect of WT and KQ Klf5 overexpression on the repression of pluripotency gene transcription by TGF-β signaling. Cells were treated as described in (B), and harvested for RNA purification and quantitative RT-PCR analysis. Averages and standard deviations from three independent experiments were plotted.

We have shown the function of Klf5 acetylation in mouse ESCs, how Klf5 acetylation is regulated in ESCs remains to be explored. It has been demonstrated that TGF-β signaling induces acetylation of KLF5 in cultured noncancerous epithelial cells, as well as prostate cancer cell lines [20, 29]. To test whether TGF-β signaling also regulates Klf5 acetylation in ESCs, we treated V6.5 ESCs with TGF-β or a TGF-β inhibitor SB525334. When TGF-β was added, the acetylation level of Klf5 is markedly increased, while other pluripotency factors, including Klf5 itself, do not show any significant changes, regardless in the presence or absence of LIF. Conversely, the treatment with SB525334 suppresses Klf5 acetylation, while total Klf5 expression level remains stable. Inhibition of TGF-β signaling also down-regulates the expression of pluripotency factors, Oct4, Sox2, and Nanog (Fig 3A). These data suggest that TGF-β signaling promotes Klf5 acetylation in ESCs, and that TGF-β signaling is involved in maintaining the expression of pluripotency genes in ESCs.

To address whether TGF-β signaling activates the expression of Oct4, Sox2, and Nanog through acetylation of Klf5, WT and KQ Klf5 were overexpressed in V6.5 ESCs, and the cells were treated with SB525334. Overexpression of WT Klf5 does not affect the down-regulation of Oct4, Sox2, and Nanog proteins caused by inhibition of TGF-β signaling. However, overexpression of KQ Klf5 prevents the suppression of Sox2 protein by SB525334 treatment, while the expression of Oct4 and Nanog still decrease upon inhibition of TGF-β signaling (Fig 3B). These data indicate that TGF-β signaling promotes Klf5 acetylation to enhance Sox2 expression. We then asked whether acetylated Klf5 regulates *Sox2* expression at the transcription level or at the post-transcriptional steps. The expression levels of *Oct4*, *Sox2*, and *Nanog* mRNA were analyzed in ESCs overexpressing WT and KQ Klf5, with or without SB525334 treatment. Consistent with Western blot result, in control ESCs, inhibition of TGF-β signaling suppresses the mRNA levels of *Oct4*, *Sox2*, and *Nanog*. The downregulation of *Nanog* mRNA, but not *Oct4*, by SB525334 treatment, can be prevented by both WT and KQ Klf5 overexpression. WT Klf5 appears to be more potent in maintaining *Sox2* mRNA expression than KQ Klf5, when TGF-β signaling is inhibited (Fig 3C). These data suggest that acetylated Klf5 enhances *Sox2* expression post-transcriptionally.

### Acetylated Klf5 suppresses differentiation genes, but activates *Cdx2*, during EB differentiation

**Fig 4:**
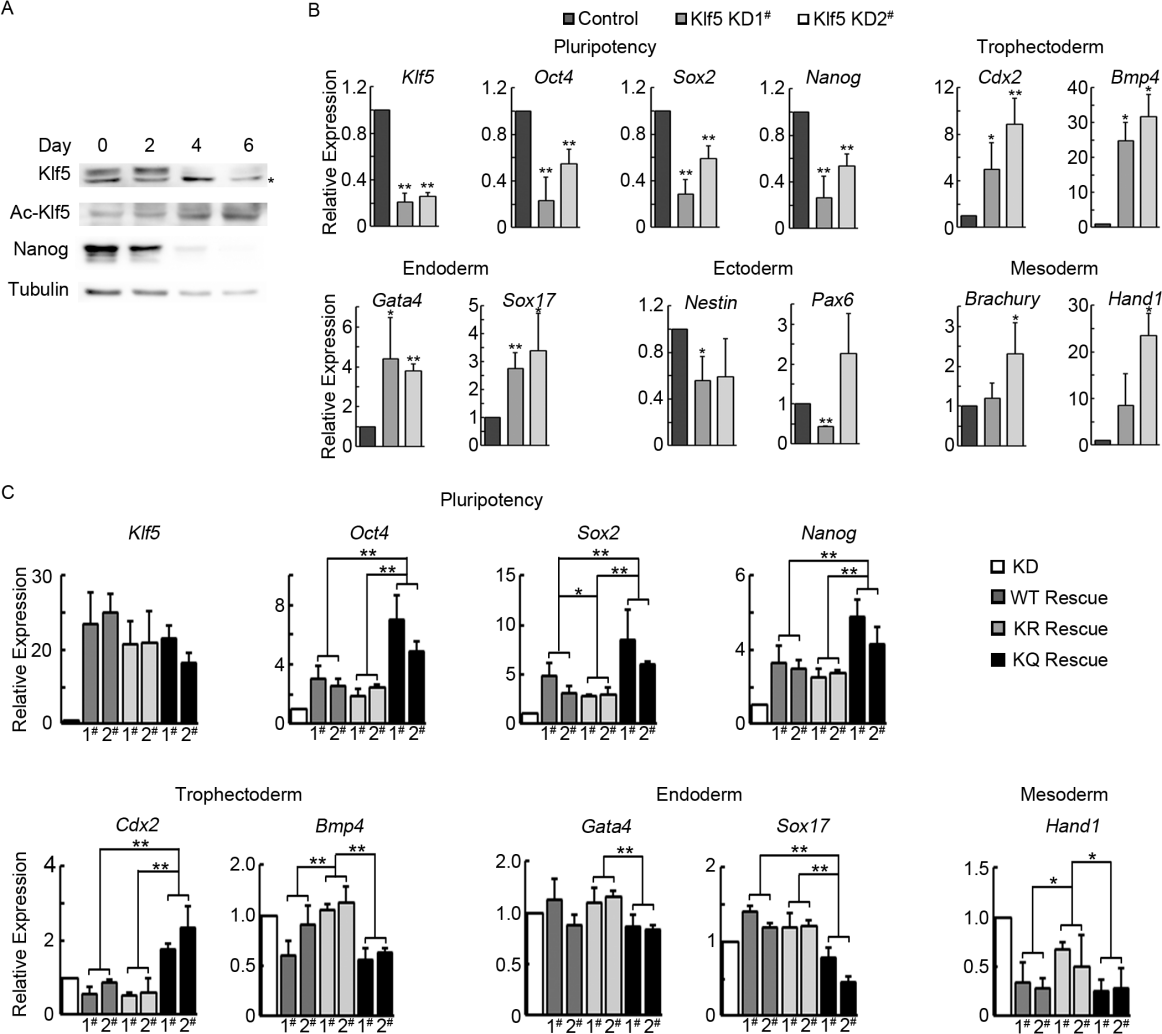
Functions of acetylated Klf5 in mouse ESC differentiation. (A) Expression of total, Ac-, and unAc-Klf5 during ESC differentiation. The nonspecific band in the anti-Klf5 blot is marked by an asterisk. V6.5 ESCs were cultured without LIF and feeder cells. Cells were harvested on day 0, 2, 4, and 6, and subjected to Western blot assay. (B) Klf5 knockdown affects the expression of pluripotency genes and differentiation genes in day 4 EBs. Day 4 EBs from shGFP control ESCs and two independent stable Klf5 knockdown ESC clones were harvested for quantitative RT-PCR analysis. Averages and standard deviations from three independent experiments were plotted. (C) Rescue effect of WT, KR, and KQ Klf5 in *Klf5* knockdown ESCs. *shKlf5* targeting sequences in WT, KR, and KQ Klf5 were mutated to render them resistant to *shKlf5* knockdown. Stable ESC lines overexpressing shKlf5-immune WT, KR, and KQ Klf5 were established in the Klf5 knockdown ESCs. Day 4 EBs from these ESCs were harvested for quantitative RT-PCR analysis. Averages and standard deviations from three independent experiments were plotted. P values were calculated by reusable two-factor analysis of variance.

It has been shown that Klf5 is involved in the lineage specification, including the ICM, the TE, and the primitive endoderm, in preimplantation embryos [14, 19]. Moreover, Klf5 suppresses mouse ESC differentiation to the mesodermal lineage [18]. Does acetylation of Klf5 play a role in regulating ESC differentiation? To address this question, we examined the expression of Klf5 and acetylated Klf5 during spontaneous ESC differentiation by LIF withdrawn. As ESCs differentiate, Klf5 expression decreases, similar to Nanog, while ac-Klf5 remains stable, and even a slightly increase on day 4 and 6 (Fig 4A). These data imply that ac-Klf5 might be involved in regulating ESC differentiation. In order to study the function of Klf5 acetylation in ESC differentiation, we constructed stable ESC lines in which endogenous *Klf5* was knocked down by shRNA, and *shKlf5*-immune WT, KQ, or KR Klf5 was overexpressed. Knockdown of *Klf5* leads to abnormal expression of pluripotency genes and differentiation genes in day 4 EBs. Pluripotency genes, *Oct4*, *Sox2*, and *Nanog*, in day 4 *shKlf5* EBs are expressed at lower levels, than those in day 4 *shGFP* EBs. Klf5 knockdown increases the expression of trophectodermal (*Cdx2* and *Bmp4*), endodermal (*Gata4* and *Sox17*) and mesodermal markers (*Brachury* and *Hand1*) in day 4 EBs, while ectodermal markers, *Nestin* and *Pax6*, are not significantly affected by downregulation of *Klf5* (Fig 4B). When WT, KR, and KQ Klf5 were overexpressed to rescue the EB differentiation abnormality caused by *Klf5* knockdown, KQ Klf5 outperforms WT and KR Klf5 in maintaining the expression of pluripotency genes. However, KQ and WT Klf5 have similar functions in suppressing differentiation genes in EBs, except for *Cdx2* and *Sox17*, while KR Klf5 has negligible effect in differentiation gene suppression. Overexpression of WT and KR Klf5 do not affect the expression of *Cdx2* and *Sox17*, while KQ Klf5 activates *Cdx2* and represses *Sox17* in EBs (Fig 4C). All these data suggest that acetylation of Klf5 is essential for its transcriptional activity in differentiated cells.

## Discussion

Klf5 is not only required for the development of the ICM, but also for the formation of the functional TE [14, 19]. In these two distinct types of cells, certain genes regulated by Klf5 should be expressed differentially. For example, *Cdx2* is activated in TE cells, and repressed in ICM cells, while the expression of *Oct4* and *Nanog* are restricted to the ICM [31–34]. How does Klf5 reverse its transcriptional activity in ICM and TE cells? The opposing functions of unAc-KLF5 and Ac-KLF5 in epidermal cells and prostate cancer cells led us to hypothesize that acetylation of Klf5 may switch the transcriptional output during the segregation of the ICM and the TE. However, when we examined the expression of total, Ac-, and unAc-Klf5 in the blastocyst, no biased expression patterns of all types of Klf5 were observed. Rather, total, Ac-, and unAc-Klf5 are evenly expressed in the TE and the ICM. Therefore, the opposing transcriptional output is not regulated by acetylation of Klf5. Other post-transcriptional modifications of Klf5 might account for the reversed transcriptional activity of Klf5 in the TE and the ICM. It has been shown that in C2C12 cells, sumoylated KLF5 interacts with unliganded peroxisome proliferator-activated receptor-δ (PPAR-δ) and co-repressors to form transcriptionally repressive regulatory complexes, whereas unsumoylated KLF5 is associated with liganded PPAR-δ and CBP to activate gene expression [27]. Alternatively, the transcriptional activity of Klf5 might be controlled by different transcription factors and regulators available in TE and ICM cells.

Acetylation of Klf5 is detectable in mouse ESCs. In contrast to KLF5 acetylation in epidermal cells and prostate cancer cells, acetylation of Klf5 in ESCs only enhances its transcriptional activity, rather than switching from a transcriptional repressor to an activator. KQ Klf5, mimicking the acetylated status, is more potent than the unacetylated KR Klf5 mutant, in activating pluripotency genes, stimulating the proliferation and self-renewal of ESCs, and reprogramming somatic cells. Despite the functional difference, acetylation of Klf5 is regulated by TGF-β signaling in ESCs, as well as in epidermal cells and prostate cancer cells [20, 29]. Interestingly, overexpression of KQ Klf5 antagonizes the downregulation of Sox2 protein by inhibition of TGF-β. It is possible that Ac-Klf5 might form a complex with Sox2 protein, and stabilize Sox2 protein. Another possibility is that a downstream target gene activated by Ac-Klf5 might enhance Sox2 protein stability. How KQ Klf5 stabilizes Sox2 protein needs to be further investigated.

During ESC differentiation, Ac-Klf5 remains stable, even slightly elevated, while total Klf5 decreases, suggesting that the fraction of Klf5 being acetylated is increased. Again, acetylation of Klf5 enhances its transcriptional activity, regardless being an activator for pluripotency genes, or being a repressor for differentiation genes. The regulation of *Cdx2* by Klf5 is different from other differentiation genes. In contrast to other differentiation genes, *Cdx2* is activated by KQ Klf5, consistent with the *in vivo* data that Klf5 is required for *Cdx2* expression in TE cells [14]. In TE cells, Klf5, together with other transcription factors, might form a transcriptional activation complex to stimulate *Cdx2* expression. In other lineages of differentiated cells, Klf5 might interact with a distinct set of transcription factors to repress transcription. Alternatively, it is also possible that Klf5 is a strong activator for *Cdx2*, and that the up-regulation of other differentiation genes is due to the down-regulation of pluripotency genes upon Klf5 knockdown. WT and KR Klf5 fail to activate *Cdx2* in EBs, implying that Klf5 acetylation might be necessary to activate *Cdx2* in TE cells.

In summary, our study elucidates the function of Klf5 acetylation in embryo development, ESC self-renewal and differentiation. In these biological processes, acetylation of Klf5 increases its transcriptional activity, rather than switching from a repressor to an activator. TGF-β signaling promotes acetylation of Klf5. Detailed mechanisms for acetylation and deacetylation of Klf5 require further investigation.

## Supporting Information

S1 Fig Confirmation of the overexpression of WT, KR and KQ Klf5 in overexpression clones. Cell lysates from WT, KR and KQ Klf5 stable overexpression clones were subjected to Western blot. The clone 1# and 2# of WT, KR and KQ overexpression clones were used in Fig 2C–2E.
(TIF)

S1 Table Primer sequences used in this study. (DOC)
