# Supplementary material for: Roles of Klf5 Acetylation in the Self-Renewal and the Differentiation of Mouse Embryonic Stem Cells
Source: PLoS One. 2015 Sep 15;10(9):e0138168. doi: 10.1371/journal.pone.0138168 (PMC4570665; doi:10.1371/journal.pone.0138168)
Supplement: S1 Table — (DOC) [file pone.0138168.s002.doc]

S1 Table. Primer sequences used in this study

| Group | Name | Sequence |
| --- | --- | --- |
| Molecular cloning | Klf5-F | GTACGGATCCATGCCCACGCGGGTGCTGA |
|  | Klf5-R | GTACGCGGCCGCTCAGTTCTGGTGGCGCTT CATGTG |
|  | Klf5-KR mut-F | GAGTAACCCGGATCTGGAGAGGCGACGTAT |
|  | Klf5-KR mut-R | CTCTCCAGATCCGGGTTACTCCTTCTGTTG |
|  | Klf5-KQ mut-F | GAGTAACCCGGATCTGGAGCAGCGACGTAT |
|  | Klf5-KQ mut-R | GCTCCAGATCCGGGTTACTCCTTCTGTTGT |
|  | Klf5-shRNA mut-Round1-F | TCTACAAATCCCAGAGGCCTTGTGTTACAC AGATCA |
|  | Klf5-shRNA mut-Round1-R | AGGCCTCTGGGATTTGTAGAGGCCAGTTCT CAGGTG |
|  | Klf5-shRNA mut-Round2-F | TCTACAAATCCCAGAGGCCTTGTGTTACGC AAATCAAGACAGAACCTGTTAC |
|  | Klf5-shRNA mut-Round2-R | TTGCGTAACACAAGGCCTCTGGGATTTGTA GAGGCCAGTTCTC |
| qPCR | Klf5-RT-F | CCGGAGACGATCTGAAACAC |
|  | Klf5-RT-R | GAAATTATCGGAACTGGAGGGA |
|  | Oct4-RT-F | ATCAGCTTGGGCTAGAGAAGGATG |
|  | Oct4-RT-R | AAAGGTGTCCCTGTAGCCTCATAC |
|  | Sox2-RT-F | GCGGAGTGGAAACTTTTGTCC |
|  | Sox2-RT-R | CGGGAAGCGTGTACTTATCCTT |
|  | Nanog-RT-F | TACAAGGGTCTGCTACTGAGATGC |
|  | Nanog-RT-R | TTGGGACTGGTAGAAGAATCAGGG |
|  | Cdx2-RT-F | CAGTCCCTAGGAAGCCAAGTGAAA |
|  | Cdx2-RT-R | AAGTGAAACTCCTTCTCCAGCTCC |
|  | Bmp4-RT-F | ACAGCGGTCCAGGAAGAAGAAT |
|  | Bmp4-RT-R | TGCACAATGGCATGGTTGGT |
|  | Gata4-RT-F | GCTATGCATCTCCTGTCACTCAGA |
|  | Gata4-RT-R | CCAAGTCCGAGCAGGAATTTGAAG |
|  | Sox17-RT-F | CCCAACACTCCTCCCAAAGTATCT |
|  | Sox17-RT-R | TCTCTGTCTTCCCTGTCTTGGTTG |
|  | Nestin-RT-F | CTGGATCTGGAAGTCAACAGAGGT |
|  | Nestin-RT-R | ATCCTCAGTTTCCACTCCTGTAGC |
|  | Pax6-RT-F | TAACGGAGAAGACTCGGATGAAGC |
|  | Pax6-RT-R | GGGCAAACACATCTGGATAATGGG |
|  | Brachury-RT-F | CATCGGAACAGCTCTCCAACCTAT |
|  | Brachury-RT-R | TACCATTGCTCACAGACCAGAGAC |
|  | Hand1-RT-F | AAGGATGCACAAGCAGGTGAC |
|  | Hand1-RT-R | TTTAATCCTCTTCTCGCCGGG |
|  | β-Actin-RT-F | CAGAAGGAGATTACTGCTCTGGCT |
|  | β-Actin-RT-R | TACTCCTGCTTGCTGATCCACATC |
